# The Effect of Particulate Matter Exposure During Pregnancy on Pregnancy and Child Health Outcomes in South Asia: Protocol for an Instrumental Variable Analysis

**DOI:** 10.2196/35249

**Published:** 2022-08-10

**Authors:** Fabian Reitzug, Stephen P Luby, Hemant K Pullabhotla, Pascal Geldsetzer

**Affiliations:** 1 Big Data Institute Nuffield Department of Population Health University of Oxford Oxford United Kingdom; 2 Woods Institute for the Environment Stanford University Palo Alto, CA United States; 3 Center on Food Security and the Environment Stanford University Palo Alto, CA United States; 4 Division of Primary Care and Population Health Department of Medicine Stanford University Palo Alto, CA United States; 5 Chan Zuckerberg Biohub San Francisco, CA United States

**Keywords:** PM2.5, air pollution, fine particulate matter, birth weight, still birth, child and maternal health, Indo-Gangetic Plain, India, Pakistan, Bangladesh, Nepal

## Abstract

**Background:**

Determining the longer-term health effects of air pollution has been difficult owing to the multitude of potential confounding variables in the relationship between air pollution and health. Air pollution in many areas of South Asia is seasonal, with large spikes in particulate matter (PM) concentration occurring in the winter months. This study exploits this seasonal variation in PM concentration through a natural experiment.

**Objective:**

This project aims to determine the causal effect of PM exposure during pregnancy on pregnancy and child health outcomes.

**Methods:**

We will use an instrumental variable (IV) design whereby the estimated month of conception is our instrument for exposure to PM with a diameter less than 2.5 μm (PM2.5) during pregnancy. We will assess the plausibility of our assumption that timing of conception is exogenous with regard to our outcomes of interest and will adjust for date of monsoon onset to control for confounding variables related to harvest timing. Our outcomes are 1) birth weight, 2) pregnancy termination resulting in miscarriage, abortion, or still birth, 3) neonatal death, 4) infant death, and 5) child death. We will use data from the Demographic and Health Surveys (DHS) conducted in relevant regions of Bangladesh, India, Nepal, and Pakistan, along with monthly gridded data on PM2.5 concentration (0.1°×0.1° spatial resolution), precipitation data (0.5°×0.5° resolution), temperature data (0.5°×0.5°), and agricultural land use data (0.1°×0.1° resolution).

**Results:**

Data access to relevant DHSs was granted on June 6, 2021 for India, Nepal, Bangladesh, August 24, 2021 for Pakistan, and June 19 2022 for the latest DHS from India.

**Conclusions:**

If the assumptions for a causal interpretation of our instrumental variable analysis are met, this analysis will provide important causal evidence on the maternal and child health effects of PM2.5 exposure during pregnancy. This evidence is important to inform personal behavior and interventions, such as the adoption of indoor air filtration during pregnancy as well as environmental and health policy.

**International Registered Report Identifier (IRRID):**

DERR1-10.2196/35249

## Introduction

South Asia is experiencing some of the highest levels of ambient air pollution globally. In cross-country comparisons of fine particulate matter with a diameter less than 2.5 μm (PM2.5), Bangladesh has emerged as the country with the highest population-weighted levels, Pakistan has the second highest levels, India has the third highest levels, and Nepal has the 12th highest levels [1]. The elevated levels of air pollution and the large number of exposed people result in significant air pollution–related mortality and morbidity in South Asia. Overall, 26.2% of all disease-adjusted life years lost to air pollution globally are estimated to occur in India alone [2]. In 2019, a total of 980,000 deaths in India, 114,000 deaths in Pakistan, 74,000 deaths in Bangladesh, and 18,000 deaths in Nepal were attributable to air pollution [3].

Our study focuses on air pollution in the Indo-Gangetic Plain (IGP), which stretches across Bangladesh, Nepal, India, and Pakistan. Pollution levels in the IGP are even higher than those in the study region in general. For instance, in the Indian part of the IGP, the annual mean PM2.5 concentration is above 100 μg/m^3^ [2], which is higher than that in India overall (83 μg/m^3^) [4], and more than 10-fold the World Health Organization’s (WHO’s) recommended limit for healthy air (10 μg/m^3^) [5]. High population density, agricultural and industrial activities, and dispersal of urban pollution to nearby rural areas, and vice versa, lead to air pollution being a public health problem in both urban and rural parts of the IGP. Moreover, rural areas considerably contribute to pollution levels through local wheat and rice stubble burning, municipal waste burning, forest fires, coal-fired factories, and other sources of rural emissions [3,6]. In the peak fire season, the mean relative effects of rural biomass burning are estimated at 30% of emission levels measured in Delhi [7]. Substantial mortality and morbidity occur in the IGP, and the number of air pollution–related deaths is approximately equally divided between urban and rural areas [3,8].

Annual mean PM2.5 levels disguise substantial seasonal variation in pollution in the IGP. While the winter months of October-February are characterized by high pollution reaching levels of over 150 μg/m^3^, pollution during the monsoon period is mostly around 50 μg/m^3^, and even heavily polluted cities such as Delhi occasionally record levels below 30 μg/m^3^ [9,10].

Seasonal variation in PM2.5 levels is particularly salient for the study of effects of pollution on pregnancy outcomes. Depending on the month of conception, fetuses experience very different levels of in utero exposure, which affects birth and child health outcomes. Moreover, exploiting seasonal variation provides a potent research design for identifying the causal effects of prenatal pollution exposure.

By now, it is well established that exposure to increased PM2.5 levels during the prenatal period is associated with a range of negative child health outcomes. Air pollution has been linked to preterm birth [11-13], low birth weight [11,14-16], increased risk of pregnancy loss and stillbirth [13,17], and longer-term developmental effects such as lower height for age [18,19].

However, the associations between negative child health outcomes and particulate matter have not been consistent across the literature. A 2017 systematic review did not find clear evidence of an association with the risk of preterm birth or term low birth weight [20], while another recent systematic review found that PM2.5 levels and low birth weight were associated in 25 of 29 studies [21]. There is also disagreement regarding the most critical pregnancy period. Some studies highlight the importance of the late pregnancy period for birth weight [11,14,22,23], while other studies found no difference [16,21].

Our study furthers the understanding of PM2.5 on pregnancy and child health outcomes in South Asia. Our study aims to (1) determine whether exposure to higher PM2.5 concentrations during pregnancy reduces the birth weight of the child; (2) determine whether exposure to higher PM2.5 concentrations during pregnancy increases the risk of neonatal, infant, and child death; (3) determine whether exposure to higher PM2.5 concentrations during pregnancy increases the risk of a pregnancy terminating in a miscarriage, abortion, or still birth; and (4) understand in which trimesters of pregnancy PM2.5 exposure most strongly reduces birth weight and increases the risk of neonatal, infant, and child death.

## Methods

### Data

The primary data set for this observational study is the Demographic and Health Surveys (DHSs) conducted in Bangladesh, India, Nepal, and Pakistan [24]. The DHSs are large, representative, cross-sectional household surveys, which include questions on topics related to health, nutrition, and demographics. Households are sampled using probability sampling based on existing sampling frames, such as a census. For each of the countries, we include all DHS waves that collected survey cluster GPS coordinates. This includes the DHS waves, which took place in Bangladesh in 2004, 2007, 2011, and 2017 to 2018; in India in 2015 to 2016 and 2019 to 2021; in Nepal in 2001, 2006, 2011, and 2016; and in Pakistan in 2006 to 2007 and 2017 to 2018. We use the women’s module, which contains information on child births, birth outcomes, maternal health, and infant mortality. We will match the DHS data (using the GPS coordinates of the survey cluster locations) to PM2.5 data to obtain our measure of prenatal pollution exposure. We will use monthly PM2.5 emissions data from the Atmospheric Composition Analysis Group at Washington University, St. Louis, Missouri, United States [25]. Monthly precipitation and temperature data are obtained from the Climate Research Unit gridded Time Series monthly high-resolution gridded multivariate climate data set with a 0.5°×0.5°–gridded resolution, published by the University of East Anglia’s Climatic Research Unit, Norwich, United Kingdom. Daily precipitation data at 0.5°×0.5°–gridded resolution is provided by the National Oceanic and Atmospheric Administration/Oceanic and Atmospheric Research/Earth System Research Laboratories Physical Sciences Laboratory, Boulder, Colorado, United States [26]. Crop data at 0.1°×0.1°–gridded resolution are obtained from the Spatial Production Allocation Model, developed by the International Food Policy Research Institute, Washington, DC, United States [27]. We assessed pregnancies having taking place between January 1998 and December 2019 since this is the period where gridded monthly air pollution data are available.

### Data Access

Access to the DHS is public but needs to be requested. We obtained access to the DHS data on June 6, 2021 for India, Nepal, and Bangladesh and on August 24 for Pakistan.

### Ethical Considerations

This research is a secondary data analysis of fully anonymized data and does not require ethics approval, as per University of Oxford institutional research ethics policy [28].

### Codebook

The DHS codebook (recode manual) provides details on the file structure, computation of additional variables, and in-depth descriptions of all variables contained in the data sets [29].

### Variables

#### Creating the Date of Conception and PM2.5 Exposure Variables

We impute the date of conception for all births within the last 5 years by subtracting the duration of an average pregnancy (40 weeks) from the date of birth reported in the DHS. The instrumental variable we construct is month of conception, a factor variable (1-12) indicating the calendar month when the pregnancy began. We also generate similar variables for each trimester of the pregnancy, using analogous procedures and assuming a length of 12.33 weeks (ie, one-third of 40 weeks) for each trimester.

Prenatal PM2.5 exposure will be computed by matching the respondents’ cluster’s GPS locations with gridded pollution data to obtain location-specific estimates of ambient PM2.5 exposure during each pregnancy. The computed measures include the following:

- Mean PM2.5 exposure during the pregnancy, measured in units of 10 μg/m^3^ (weighted)
- Median PM2.5 exposure during the pregnancy, measured in units of 10 μg/m^3^ (including also partial months)
- 10th, 25th and 75th, and 90th percentile monthly PM2.5 exposure during pregnancy, measured in units of 10 μg/m^3^ (including also partial months)
- Maximum monthly PM2.5 exposure during pregnancy, measured in units of 10 μg/m^3^ (including also partial months)
- Cumulative PM2.5 exposure over the whole pregnancy period, measured in units of 10 μg/m^3^ (weighted)
- Number of high or low PM2.5 exposure months over the whole pregnancy period (number of months above or below mean PM2.5 levels at the respondent’s location, including partial months)
- Mean PM2.5 exposure over the whole pregnancy period relative to the annual location-specific average, measured in units of 10 μg/m^3^ (weighted)

All exposures with “weighted” in brackets are weighted by the fraction of the month that the pregnancy covers (between 0 and 1). For median, percentiles, minimum, and maximum exposure partial months are included.

For each trimester of the pregnancy, the following measures are computed:

- Mean PM2.5 exposure during each trimester of the pregnancy, measured in units of 10 μg/m^3^ (weighted)
- Cumulative PM2.5 exposure over each trimester of the pregnancy, measured in units of 10 μg/m^3^ (weighted)
- Number of high or low PM2.5 exposure months over the whole pregnancy period (number of months above or below mean PM2.5 levels at the respondent’s location, including partial months)
- Mean PM2.5 exposure over the whole pregnancy period relative to the annual location-specific average, measured in units of 10 μg/m^3^ (weighted)

#### Creating the Environmental Covariates

Using gridded temperature and precipitation data, we compute the mean temperature and precipitation for each trimester of the pregnancy, the mean over the whole pregnancy, and the mean over the neonatal period, infancy, and childhood.

We generate a variable for monsoon onset to account for spatiotemporal variation in weather patterns that could influence the timing of conception, pollution, and harvest. Using daily precipitation data and a previously validated method [30], we generate a year-specific variable (taking values from 1-365 or 1-366 in a leap year), which indicates the date of local monsoon onset in the year of conception.

#### Outcome Variables

##### Birth Weight

The following outcome variables will be considered:

1. Birth weight (in g, m19; primary outcome)
2. Low birth weight (<2500 g, m19; secondary outcome)
3. Very low birth weight (<1500 g, m19; secondary outcome)
4. Extremely low birth weight (<1000 g, m19; secondary outcome)

Birth weight (in g) is a continuous variable, and the additional aforementioned birth weight variables (2)-(4) are binary variables derived from the continuous variable. We generate binary variables for *low*, *very low*, or *extremely* in the following way: low birth weight is defined as weight<2500 g, very low birth weight as weight<1500 g, and extremely low birth weight as weight<1000 g (according to the WHO’ definition [31]).

Note that all variables referenced by the short variable name (m19, etc) are directly from the DHS data set. All other variables are imputed or are obtained from additional non-DHS data sets.

##### Miscarriage, Abortion, or Still Birth

The rate of pregnancy termination resulting in miscarriage, abortion, or still birth per 1000 pregnancies (based on v229, v230, and v233) is the primary outcome.

Pregnancy termination will be calculated as a rate per 1000 pregnancies (and PM2.5 exposure of these pregnancies will be computed from the imputed conception date to the point of pregnancy termination).

##### Neonatal, Infant, and Child Mortality

The following outcome variables will be considered:

1. Neonatal death rate per 1000 children (age 28 days, b5; primary outcome)
2. Infant death rate per 1000 children (age 1, b5; primary outcome)
3. Child death rate per 1000 children (age 5, b5; primary outcome)

#### Explanatory Variables and Covariates

##### Explanatory Variables (PM2.5 Concentration)

The following explanatory variables will be considered:

1. Mean PM2.5 exposure during the pregnancy (weighted)
2. Median PM2.5 exposure during the pregnancy (including also partial months)
3. 10th, 25th and 75th, and 90th percentile monthly PM2.5 exposure during pregnancy (including also partial months)
4. Maximum monthly PM2.5 exposure during pregnancy (including also partial months)
5. Cumulative PM2.5 exposure over the whole pregnancy period (weighted)
6. Number of high or low PM2.5 exposure months over the whole pregnancy period, including partial months
7. Mean PM2.5 exposure over the whole pregnancy period relative to the annual location-specific average (weighted)
8. All of the above PM2.5 variables, but computed separately for each trimester of the pregnancy

PM2.5 exposure is a continuous variable (our instrument) that is created by using the month of conception to instrument for the exposure during each trimester or the whole pregnancy (and controlling for additional covariates described in the analysis section).

##### Covariates for the Main Models

Following are the covariates for the main models:

1. Preceding birth interval
2. Birth order (hwidx)
3. Maternal age at birth of the child
4. Sex of the child (b4)
5. Twin birth
6. Mean temperature during the pregnancy or for each trimester
7. Mean precipitation during the pregnancy or for each trimester
8. Monsoon onset
9. Percentage of land used for rice cultivation

Preceding birth interval is an unordered factor variable that indicates the time difference in months between the current and the previous birth (categories: ≤12 months, >12 and ≤24 months, >24 and ≤36 months, and >36 months). Birth order is a factor variable where 1 indicates the most recent birth. Maternal age provides the age (in years) of the respondent at each of child birth in the data set. Sex of the child is a binary variable. Twin birth is a binary variable. Mean temperature and precipitation are derived from gridded monthly values at the respondent’s location and averaged over the pregnancy period. Monsoon onset is a discrete variable (1-365/366) indicating the day of the year when the monsoon starts. Rice fraction is a continuous variable (0-1) for the percentage of land used for rice cultivation at the respondent’s location.

##### Additional Covariates for Some of the Robustness Checks

The following are additional covariates for robustness checks:

1. Respondent's education level (v106/v133)
2. Wealth index (v190/v191)
3. Respondent's age (v012)
4. Respondent’s height (v438)
5. Altitude (v040)
6. Religion (v130)
7. Ethnicity or caste (v131)
8. Marital status (v501)
9. Region and primary sampling unit (DHSREGNA, DHSCLUST)
10. Type of cooking fuel used in the household (v161)
11. Total number of children ever born (v201)
12. Antenatal care visit in the first trimester (m13)
13. Received toxoid injections during pregnancy (m1)
14. Took iron tablets or syrup during pregnancy (m45)
15. Took antimalarial drugs (SP/Fansidar or Chloroquine, m49a-m49b)
16. Husband’s occupation (v705)
17. Smoking (v464)

Education and the wealth indicators are available both as categorical and continuous variables, age, height (in cm), and altitude (in m) are continuous. Religion and ethnicity or caste are factor variables with country-specific levels. Marital status is a factor variable. Region and primary sampling unit are factor variables, and cooking fuel is a proxy for indoor air pollution and is a factor variable indicating the type of fuel used for cooking inside the house. Number of births is continuous. Antenatal care visit is a binary variable indicating whether a care visit took place in the first trimester. The variables for toxicoid injections, antimalarial drugs, and iron tablets or syrups are binary variables indicating whether the respondent received these medicines during pregnancy. Husband’s occupation is a factor variable with standardized categories across countries. Smoking is a continuous variable indicating the number of cigarettes smoked in the last 24 hours by the respondent.

#### Unit of Analysis

We have restricted our sample to mothers who have children born within the last 5 years (v208). This is because day of birth (hw16) is only recorded for these children, which gives us more precision for our exposure estimates (we also exclude all births where the date of birth was missing, except for all children who died after birth where the date of birth was not recorded). We further restrict the sample to all mothers with at least 2 births within the last 5 years to exploit variation in timing of multiple births from the same mothers. This allows us to control for observed and unobserved individual differences between mothers. We also exclude all respondents where de facto and de jure regions of residency differed.

### Statistical Analyses

#### Data Exclusion

DHS surveys are cleaned by a professional team, and we do not expect many outliers. In some questions, implausible values are already flagged in the data; for instance, if the recorded age of death is a time point after the date of the interview, the value is flagged in B13. In such instances, we will not include these data points in our analysis.

#### Power

We have not assessed statistical power to detect our minimum effect sizes. We will conclude that the analysis supports our hypotheses if both the effect sizes are larger than the minimum effects specified under “effect sizes” below, and the respective *P* values are <.05.

#### Statistical Models

The following is an overview of our analytic strategy:

1. The sample will be restricted to respondents with multiple births, who reside in the IGP.
2. The IVs (joint estimation of the first and second stage) will be run: use of the month of conception to instrument for PM2.5 exposure during pregnancy; use of instrument and covariates to predict pregnancy and child health outcomes
3. Robustness checks: is the exclusion restriction plausible, that, after adjusting for all time-invariant confounders at the level of the mother (through mother-level fixed effects) as well as temperature and precipitation during the pregnancy period, the month of conception affects birth outcomes through no channel other than PM2.5 exposure?

Details of the analysis steps are as follows:

1. We include all respondents in Bangladesh, Nepal, Pakistan, and India whose survey cluster GPS coordinates fall within the boundaries of the IGP. The boundaries of the IGP were obtained from a previous study [32]. We also have restricted the sample to mothers with multiple births. By exploiting within-mother variation in the timing of births and using mother–fixed effects, we can more plausibly account for (un)observable mother-level factors that may otherwise confound our estimates.
2. We will estimate our models with an instrumental variables (IV) estimator using the R package fixest. We will use interactions of month of conception indicators with regional fixed effects to instrument for location-specific effects of month of conception on PM2.5 exposure during pregnancy (and alternatively each semester of the pregnancy). Modeling location-specific effects of month of conception on PM 2.5 exposure is important to satisfy the monotonicity assumption required of the IV estimator. We have defined regions on the basis of level 2 administrative boundaries, and we will check for robustness using other definitions of regions. We will also control for the date of the monsoon onset in the year of conception (as well as the interaction between monsoon onset and the share of rice grown at the respondents’ location). The second stage of the IV estimation uses the predicted PM 2.5 values from the first stage to estimate the effect of PM 2.5 exposure on our outcomes. Our regression model includes mother fixed effects, which account for any time-invariant mother-specific unobservable factors. To account for aggregate time-varying factors, we will include birth year fixed effects. Our specification controls for temperature and precipitation, which are observable factors that may be correlated with both the month of conception and birth weight. We also have included birth-level covariates that correlate with birth outcomes, including preceding birth interval, birth order, maternal age at the birth of each child, twin birth, and sex of the child. We have 3 sets of outcomes:
  - Birth weight: a continuous variable (primary outcome) and binary outcomes low, very low, or extremely low birth weight (secondary outcome).
  - Miscarriage, abortion, or still birth: pregnancies terminating in the first, second, and third trimesters (we restricted the sample to all women who reported a pregnancy ending in miscarriage, abortion, or still birth). These are provided as rates per 1000 births.
  - Neonatal, infant, and child mortality: outcomes for neonatal, infant, and child death. These are provided as rates per 1000 children.
3. We will use multiple tests to assess the robustness of our estimates and the plausibility of the exclusion restriction. To test for differences between subgroups, we will rerun the main models as follows: (1) by including only urban or rural respondents (since urban respondents may be less affected by seasonality, whereas rural respondents’ lifestyle and nutrition may depend more on agriculture, weather conditions, etc., v102), (2) by including only households working in agricultural or nonagricultural professions (since rural households working in agriculture may experience more seasonality in their lifestyles than nonagricultural households, v716), (3) including only wanted or unwanted pregnancies (v367) since desired and undesired pregnancies may have different seasonal patterns. We will also run the models without restricting ourselves to mothers with multiple births but including all mothers and controlling for the covariates described above (since we cannot use mother-level fixed effects in this case). Finally, we also assess how our estimates change when we use the month of birth instead of the month of conception as the instrument.

Variables that violate the assumption of homoscedasticity or of normality (as determined by a Kolmogorov-Smirnov test) will be transformed (for instance, using log-transformation). 

#### Effect Size

A recent meta-analysis reported that increased pollution is associated with an 11% increase in median risk for low birth weight (mainly from US studies) [21]; thus, we would expect an effect at least as large (since exposure in India is much higher than that reported in the studies included in the meta-analysis). For low birth weight, we have not identified such a clear minimum expected value from previous studies, but we expect significant reductions in birth weight. For infant mortality, a summary of studies from developed and developing countries reports increases in mortality ranging from 10%-35% per 10 μg/m^3^ increase in PM2.5 concentration [12]. The lower bound of their confidence interval is 5%, which is the minimum effect size of interest for us.

#### Reliability and Robustness Testing

We will use different PM2.5 concentration measures described above to assess robustness of our findings. Since the incidence of preterm births is positively associated with pollution (but we do not know which births are preterm), we can assess the reliability of our results to assuming a more conservative, imputed pregnancy duration of <40 weeks for all pregnancies. We will draw upon regional statistics on gestational length when doing so. We may also include different constructs for measuring pollution—for example, fire counts from satellite data—to evaluate the robustness to an alternative way of capturing pollution exposure.

## Results

Data cleaning and processing have been completed. This study has also been preregistered in the Open Science Foundation registries (registration digital object identifier 10.17605/OSF.IO/TBQFH). Data analysis began in August 2021.

## Discussion

### Principal Findings

Our study will test the hypothesis that exposure to higher concentrations of PM2.5 during pregnancy reduces the birth weight of the child, and that exposure to high concentrations during pregnancy increases the risk of neonatal, infant, and child death as well as the risk of a pregnancy terminating in a miscarriage, abortion, or still birth. We will also investigate during which trimester the negative effects of PM2.5 (if any) on pregnancy outcomes are most pronounced.

### Strengths and Limitations

This study makes several contributions to the literature. First, we draw upon a novel data set with globally consistent monthly PM2.5 concentrations. The unavailability of such monthly resolved PM2.5 data sets for the IGP has thus far been an impediment to the study of health effects over the entire area. Second, while other studies have investigated effects of more short-term exposures, such as wildfires [14,33] whose duration is often in the order of days, high seasonal pollution levels in the IGP enable us to study a population with high exposures over multiple months. Third, if we can show that timing of birth affects pregnancy and child health outcomes through no channel other than PM2.5 exposure (after controlling for observable factors that could intervene in this relationship), we can interpret our findings as having causal meaning. To make this assumption plausible, we account for individual-level confounders by focusing our analysis on within-mother variation in the timing of birth and by comparing the outcomes of children born to the same mother but in different months of the year. We also account for time-varying, seasonal factors such as temperature, precipitation, and local monsoon onset.

We are aware of several factors that may limit the validity of our results. First, while we can carefully control for individual-level factors and time-varying seasonal factors we cannot exclude with certainty that our instrument (month of conception) does not influence our outcome variables through channels other than PM2.5 exposure. Second, PM2.5 exposure is variable across small geographic scales, which we cannot capture. Besides ambient air pollution, respondents may also experience indoor air pollution, which is a second important pollution source, which we cannot directly measure. However, we control for household-level factors to account for unobserved household-level confounders. Exposure measurement in our study is thus limited both by the scale of the PM2.5 data as well as the systematic random displacement of GPS coordinates in the DHS survey (used to deidentify respondent’s locations). Third, we infer the month of conception based on the duration of an average pregnancy (40 weeks). However, we do not know the actual length of the pregnancy periods of our respondents, which may lead to incorrect estimation of PM2.5 exposure of the individual pregnancies. Since pollution is associated with shorter gestational period, our estimates of PM2.5 exposure are likely to be upward biased and may misclassify the trimester during which the exposure occurred. We seek to address this bias by making more conservative assumptions for an average pregnancy duration. Fourth, we cannot distinguish between preterm and term births owing to unknown length of the gestational period. Thus, we are unable to disentangle the mechanism underlying low birth weight. Low birth weight at term could be the result of PM2.5 exposure. Alternatively, PM2.5 may increase the risk of premature birth, which, on average, results in lower birth weight [34].

### Practical Significance

Air pollution is a major contributor to the global disease burden. While deaths due to indoor air pollution have been declining [35], ambient air pollution has become the fifth leading global cause of death in 2015. It is the second leading cause in India, fourth leading cause in Pakistan, and fifth leading cause in Bangladesh [36]. In the countries included in our study, population-weighted pollution levels have increased in recent years, which indicates a need to address this growing public health problem. Studies on the effects of postnatal PM2.5 exposure may not account for the fact that the same populations often already experience prenatal exposure. Our study contributes to the understanding of negative health effects of prenatal exposure and is able to disentangle the effects of prenatal exposure from the effects of postnatal exposure. By doing so, the study seeks to draw attention specifically to the effects of pollution during pregnancy and to raise awareness of potential negative pregnancy and child health outcomes.

### Future Directions

Future work should expand this study’s approach and investigate the effect of PM2.5 on pregnancy outcomes in regions other than South Asia. The use of natural experiments, such as seasonal variation in PM2.5 or discontinuities in exposure on small spatial scales, could allow researchers to provide evidence on the causal effects of PM2.5 during pregnancy in different contexts, countries, and regions. In addition, future work would benefit from the availability of more reliable measures of gestational length to obtain precise measures of length of exposure. Finally, remotely sensed PM2.5 data, which are spatially and temporally more granular and incorporate high-resolution ground measurements or measurements from wearable sensors, could provide more accurate data on respondents’ actual exposure.

## Abbreviations

DHS: Demographic and Health Surveys
IGP: Indo-Gangetic Plain
IV: instrumental variable
PM: particulate matter
PM2.5: particulate matter with a diameter less than 2.5 μm
WHO: World Health Organization
